# Surgical dilemmas in Gardner syndrome: infiltrative basal cell carcinoma and total knee prosthesis failure

**DOI:** 10.1093/jscr/rjaf285

**Published:** 2025-05-12

**Authors:** Q Carlos Diaz, Victor H Argueta, Pedro Chajon, Andrea Argueta

**Affiliations:** Universidad Francisco Marroquín, Guatemala City, Guatemala; Universidad Francisco Marroquín, Guatemala City, Guatemala; Universidad Francisco Marroquín, Guatemala City, Guatemala; Universidad Francisco Marroquín, Guatemala City, Guatemala

**Keywords:** Gardner syndrome, infiltrative basal cell carcinoma, total knee prosthesis failure, genetic disorders, orthopedic complications

## Abstract

Infiltrative basal cell carcinoma is one of the most common forms of skin cancer, generally presenting as a slow-growing, locally invasive lesion limited to the epidermis. However, in patients with genetic syndromes such as Gardner syndrome, basal cell carcinoma may present more aggressively and in atypical locations. This case describes a 52-year-old male patient with Gardner syndrome, presenting with infiltrative basal cell carcinoma on the oral mucosa and suffering from catastrophic failure of total knee prosthesis due to postoperative complications including infection and bone necrosis. The combination of these two unusual clinical factors presents a challenge for both oncological management and surgical treatment. This case highlights the complex interaction between genetic predisposition to cancer and orthopedic complications, emphasizing the need for a multidisciplinary approach.

## Introduction

Basal cell carcinoma (BCC) is the most common type of skin cancer, accounting for approximately 75% of all skin cancers in adults [1]. Its incidence increases with age and chronic sun exposure. However, in patients with genetic conditions such as Gardner syndrome, the occurrence of BCC may be more frequent and aggressive. Gardner syndrome is a rare autosomal dominant genetic disorder characterized by the presence of multiple osteomas, epidermoid cysts, fibromas, and an increased risk of developing colorectal polyps and cancer. The condition is caused by mutations in the APC gene, which plays a crucial role in cell signaling and growth. The incidence of Gardner syndrome is estimated at 1 in 100 000 to 1 in 160 000 individuals, with variable expressivity and penetrance [2].

On the other hand, total knee prosthesis is a commonly performed procedure for the treatment of severe osteoarthritis. However, prosthetic failure can occur due to various postoperative complications, including infection, mechanical overload, and poor wound healing. The coexistence of BCC and prosthetic failure in a patient with Gardner syndrome creates a highly complex clinical scenario, as both conditions require specialized management. This report presents the rare combination of these two conditions and discusses the challenges in their treatment.

## Case presentation

A 52-year-old male patient with a known history of Gardner syndrome presented with persistent pain and swelling in his left knee. Gardner syndrome was diagnosed through the detection of multiple osteomas on CT scans, a family history of colon cancer, and the presence of adenomatous polyps found during a colonoscopy. Genetic testing confirmed a mutation in the APC gene. His medical history included multiple osteomas detected on previous radiographic exams, as well as a family history of colon cancer. Due to progressive knee osteoarthritis and reduced mobility, he underwent a left total knee arthroplasty three months prior to presenting to our clinic.

**Figure 1:**
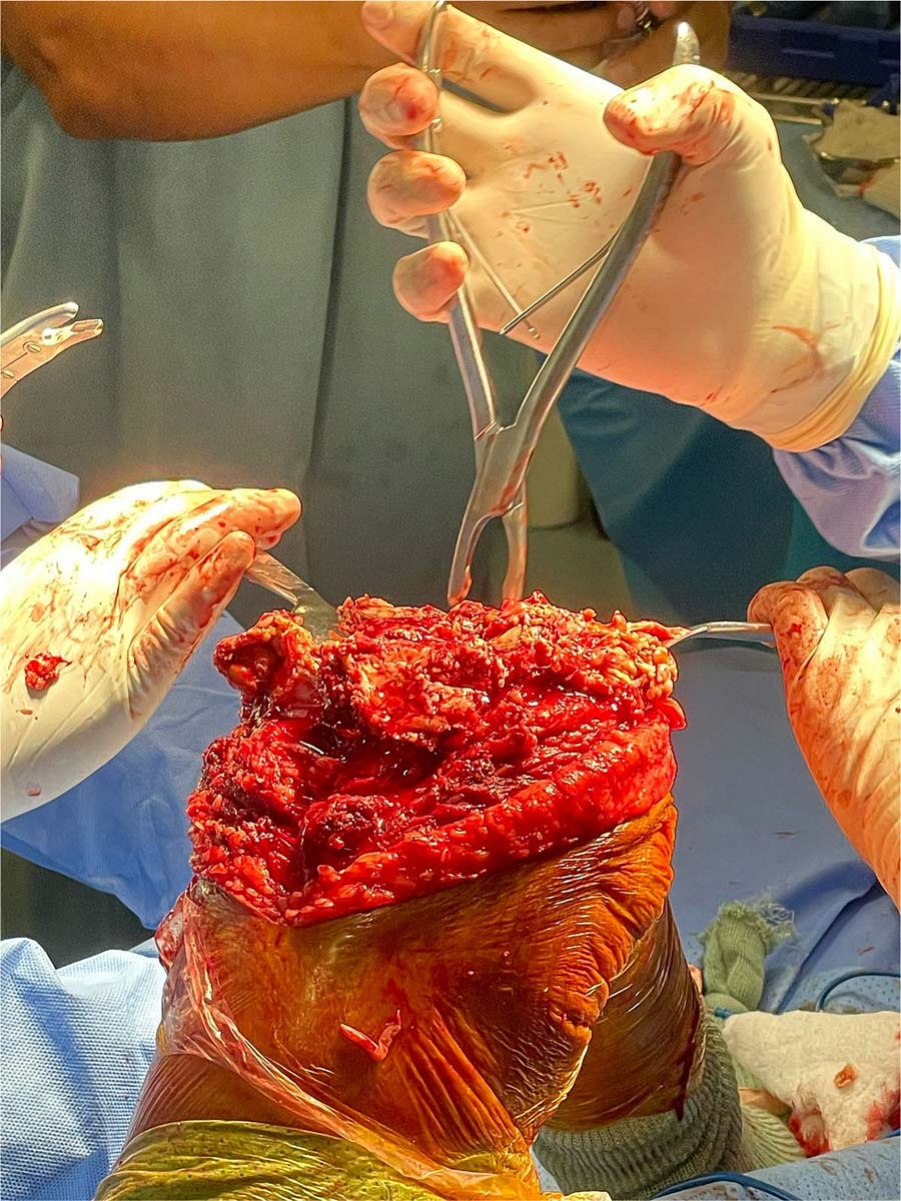
Total knee prosthesis revision surgery. This image depicts the surgical site during the revision of the left total knee prosthesis. The femoral component is seen to be loosened and fractured, with significant bone necrosis surrounding the prosthetic implant. The revision surgery involved the removal of the failed prosthesis and the use of a custom implant designed to accommodate the patient’s underlying bone abnormalities.

Postoperatively, the patient experienced delayed wound healing and an unusual discharge from the surgical site. After several weeks of conservative management, he developed severe pain, increased swelling, and signs of systemic infection. Radiographs revealed bone necrosis around the prosthetic components, and surgical exploration confirmed a catastrophic failure of the knee prosthesis, with loosening and fracture of the femoral component (Fig. 1).

**Figure 2:**
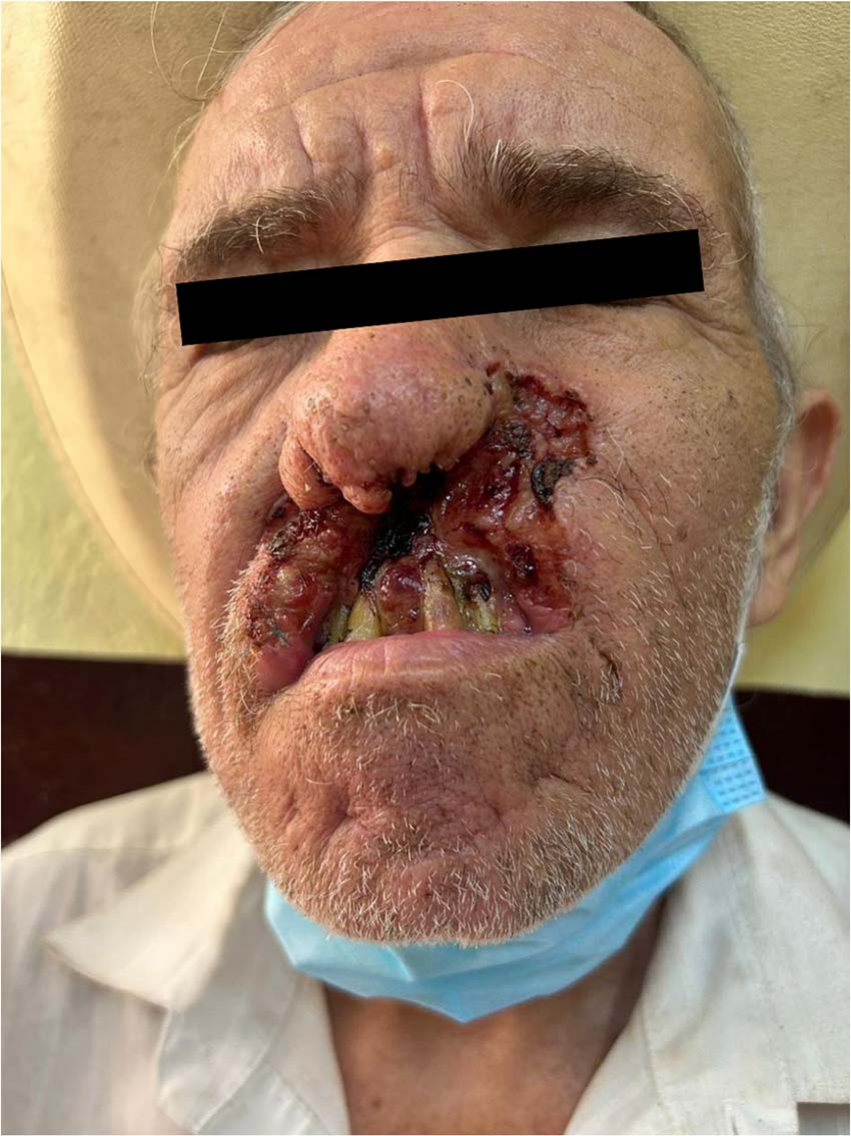
Oral mucosal infiltrative basal cell carcinoma. Image shows a biopsy-proven infiltrative basal cell carcinoma on the buccal mucosa of the patient. The lesion appears as an ulcerated, slow-growing mass with irregular borders. The presence of significant tissue invasion is evident, which led to the decision for surgical excision and radiation therapy.

Simultaneously, the patient had been noticed to develop a slow-growing lesion in his oral cavity. The lesion, initially mistaken for a benign ulcer, grew in size over several months, becoming ulcerated and painful (Fig. 2). Biopsy results confirmed the diagnosis of infiltrative BCC. Further examination revealed no other cutaneous lesions, but a thorough examination confirmed the presence of multiple osteomas in the mandible and skull, consistent with the diagnosis of Gardner syndrome.

The patient’s complex medical and surgical history necessitated a multidisciplinary approach for management. His Gardner syndrome had predisposed him to both BCC and the development of multiple osteomas, complicating the management of his knee prosthesis. The prosthetic failure required a revision surgery, while the BCC was treated with surgical excision and subsequent radiation therapy due to the high risk of local recurrence.

## Discussion

Gardner syndrome is a rare genetic disorder caused by mutations in the APC gene, leading to the formation of osteomas, epidermoid cysts, fibromas, and BCCs. BCCs in patients with Gardner syndrome tend to be more aggressive and occur earlier in life compared to the general population. The most common locations for BCCs are the head and neck regions, but in this patient, the carcinoma manifested in the oral mucosa, which is a rare presentation [3, 4].

The management of BCC typically involves surgical excision, with the goal of achieving clear margins. However, in cases where the carcinoma is infiltrative and has spread beyond the skin layers, additional treatments such as radiation therapy may be necessary. This patient’s BCC required a combination of surgical excision followed by localized radiation therapy due to its invasive nature and the high likelihood of recurrence [5]. The role of radiation therapy in the treatment of BCC, particularly in patients with genetic predispositions such as Gardner syndrome, remains an area of active research [6].

The failure of the total knee prosthesis in this patient highlights the challenges that can arise in individuals with underlying genetic syndromes [7]. Although total knee arthroplasty is generally a safe and effective procedure for patients with osteoarthritis, those with Gardner syndrome may be at higher risk for complications such as delayed wound healing, infection, and bone necrosis. The presence of osteomas can further complicate surgical procedures, as these benign bone tumors may interfere with normal bone remodeling and healing. In this patient, the failure of the knee prosthesis was attributed to both infection and mechanical overload due to the bone changes associated with his genetic condition [8].

In the context of Gardner syndrome, the combination of BCC and orthopedic complications presents a unique challenge. The patient’s care required coordinated management by both oncologists and orthopedic surgeons. Given the complexity of the case, a personalized treatment plan was essential, incorporating both oncological and orthopedic considerations.

This case underscores the importance of early recognition and comprehensive management of patients with Gardner syndrome, as they are at increased risk for multiple malignancies and orthopedic complications [9]. Furthermore, it highlights the need for careful surgical planning and postoperative monitoring, particularly in patients with genetic conditions that predispose them to abnormal bone growth and wound healing.

## Conclusion

The coexistence of infiltrative BCC and total knee prosthesis failure in a patient with Gardner syndrome is an exceptionally rare and complex clinical scenario. The aggressive nature of BCC in this patient, coupled with the orthopedic complications related to his genetic condition, presented significant management challenges. A multidisciplinary approach, involving both oncological and orthopedic expertise, was crucial for achieving the best possible outcome for this patient. This case highlights the need for clinicians to be aware of the potential for multiple, concurrent pathologies in patients with rare genetic syndromes, and the importance of personalized care in managing such complex cases.

## Data Availability

All data supporting this case report are included in the article, with no additional data available due to patient confidentiality.
